# Evaluating the implementation of *HeLP-Diabetes* within NHS services: study protocol

**DOI:** 10.1186/1472-6963-14-51

**Published:** 2014-02-04

**Authors:** Jamie Ross, Fiona Stevenson, Charlotte Dack, Kingshuk Pal, Carl May, Susan Michie, Steve Parrott, Elizabeth Murray

**Affiliations:** 1 1e-Health Unit, UCL Research Department of Primary Care & Population Health, Upper 3rd Floor, Royal Free Hospital, Rowland Hill Street, London, NW3 2PF,m, UK; 2 Faculty of Health Sciences, University of Southampton, Highfield, Southampton, SO17 1BJ, UK; 3 Centre for Outcomes Research and Effectiveness Department of Clinical, Educational and Health Psychology, University College London, 1-19 Torrington Place, London, WC1E 7HB, UK; 4 Department of Health Sciences, Seebohm Rowntree Building, University of York, York, YO10 5DD, UK

**Keywords:** Implementation, Diabetes, Health services research, Internet, Self-management

## Abstract

**Background:**

Self-management by people with type 2 diabetes is central to good health outcomes and the prevention of associated complications. Structured education to teach self-management is recommended by the National Institute for Heath and Clinical Excellence; however, only a small proportion of patients report being offered this education and even fewer attend. This study aims to evaluate the implementation of a new internet-based self-management intervention: *HeLP-Diabetes* (Healthy Living for People with type 2 Diabetes) within the National Health Service. Specific objectives are to *a)* determine the uptake and use of *HeLP-Diabetes* by services and patients; *b)* identify the factors which inhibit or facilitate use; *c)* identify the resources needed for effective implementation; *d)* explore possible effects of *HeLP-Diabetes* use on self-reported patient outcome measures.

**Methods/Design:**

This study will use an iterative design to implement *HeLP-Diabetes* into existing health services within the National Health Service. A two stage implementation process will be taken, whereby batches of General Practice surgeries and diabetes clinics will be offered *HeLP-Diabetes* and will subsequently be asked to participate in evaluating the implementation. We will collect data to describe the number of services and patients who sign up to *HeLP-Diabetes*, the types of services and patients who sign up and the implementation costs. Semi-structured interviews will be conducted with patients and health professionals and cohorts of patient participants will be asked to complete self-report measures at baseline, 3 months, and 12 months.

**Discussion:**

This study will evaluate the implementation of a new online self-management intervention and describe what happens when it is made available to existing National Health Services and patients with type 2 diabetes. We will collect data to describe the uptake and use of the intervention and the resources needed for widespread implementation. We will report on patient benefits from using *HeLP-Diabetes* and the resources needed to achieve these in routine practice. Interviews with key stake holders will identify, define and explain factors that promote or inhibit the normalization of new patterns of patient and professional activity arising from *HeLP-Diabetes*.

## Background

Diabetes is one of the most common long-term conditions in the UK affecting around 4.5 per cent of the population. It is a leading factor in the development of chronic illnesses and complications including coronary heart disease, renal failure and blindness, as well as reducing life expectancy [1]. Diabetes and related complications put considerable strain on health budgets with the estimated cost to the National Health Service (NHS) of £14 billion a year [2].

Self-management by the patient is central to good diabetes care [3-5], and structured education in which the knowledge and skills necessary for self-management are taught, has been found to provide a fourfold decrease in the risk of complications developing [6]. Structured education at diagnosis with annual reinforcement is recommended by the UK’s National Institute for Health and Clinical Excellence (NICE) for all people with type 2 diabetes (T2DM). However, only 8.7 per cent of people with T2DM who were newly diagnosed in 2008–2009 reported being offered structured education during 2009–2011 while only 3.6 per cent reported actually attending structured education [7].

Structured education, currently delivered as a group based activity, may be particularly unsuitable for people with caring responsibilities, some ethnic minority populations, those with physical disabilities and those who find group interactions challenging. With NHS resources unlikely to increase for such education and the poor referral and uptake of existing programmes there is an urgent need for new cost effective ways of delivering self-management education which are acceptable to patients and clinicians. The use of new information and communication technologies to improve health and health care is now a central part of NHS policy, and NHS Choices is already delivering a number of websites although many of these have yet to be evaluated.

The internet offers huge potential for delivering public health interventions [8]. Internet- based interventions have a number of potential benefits including convenience, accessibility, anonymity and may be cost-effective in delivering education to large numbers of people. Although the current evidence on the use of new technology in diabetes is still evolving, positive effects of such interventions have been demonstrated in several studies. A Cochrane systematic review of interactive healthcare applications looked at 24 randomised control trials (RCTs) in a range of chronic diseases including diabetes. It found mostly positive effects, with users tending to become more knowledgeable, feel better supported, with possible improved behavioural and clinical outcomes compared with non-users [9]. Another systematic review produced a narrative report of interactive computer-assisted technology in diabetes care [10]. It identified 14 studies that looked at HbA1c levels (a measure of glucose metabolism which reflects average blood glucose levels over six to eight weeks) and found that 6 of 14 studies demonstrated significant improvements in HbA1c. The interventions also appeared to improve healthcare utilisation with more foot examinations and HbA1c monitoring. Evidence from a recent systematic review of 16 RCTs suggests internet-based self-management programmes for people with T2DM can improve HbA1c [11]. This review suggested that these positive effects on HbA1c may decrease over time, which is similar to the effects of more traditional group-based self-management education on HbA1c which also show a similar decline in benefits over time [4,12,13].The American Diabetes Association standards for self-management education recognise the need for both initial training and subsequent on-going support to try and maintain the benefits of educational interventions [14]. Internet-based interventions are ideally suited to providing ongoing self-management support that could allow sustained improvements and long-term improvements in outcomes.

We have developed an internet based self-management intervention for people with T2DM: '*HeLP-Diabetes: Healthy Living for People with Type 2 Diabetes’*. The design of this complex intervention has been informed by theory and the needs and preferences of patients and health professionals. It has been developed using a process of participatory design with users (people with T2DM and health professionals) heavily involved in the conception and creation. *HeLP-Diabetes* takes a holistic view of self-management and addresses a wide range of patient needs including education, lifestyle changes, medicine management, emotional management, social support with forums and personal stories and also addresses how patients interact and work with health professionals. A facility to interface with patients electronic medical records has been designed to provide patients access to self-management metrics recorded by General Practice (GP) surgeries. The information provided on *HeLP-Diabetes* is based on NICE guidelines and has been developed to complement existing structured group education.

This study will explore the implementation of *HeLP-Diabetes* within the NHS, addressing the facilitators and barriers associated with initial and continued engagement by staff and patients and the resources required to make *HeLP-Diabetes* a viable part of routine diabetes care. This implementation study is being conducted in parallel to a randomised control trial of the *HeLP-Diabetes* intervention. The Medical Research Council (MRC) framework for complex interventions [15] stresses the importance of considering the implementation of complex interventions throughout the development and evaluation phases in a cyclical and iterative way. The data provided by this implementation study will provide additional outcome data to that provided by the trial including data that will be essential for commissioners of care such as data on what happens in practice, and what actions and resources are needed for successful implementation and realisation of potential benefits.

### Aim

To explore the implementation of *HeLP-Diabetes* within NHS GP surgeries and hospital and community based diabetes clinics.

### Objectives

a. To describe the uptake and use of *HeLP-Diabetes* by NHS services and patients.

b. To identify factors which inhibit or facilitate implementation.

c. To identify the resources needed for effective implementation.

d. To explore any effects of *HeLP-Diabetes* use on self-reported patient outcomes.

### Theoretical framework

Literature on the transfer of research into clinical practice consistently reports the process as unpredictable, slow and haphazard [16]. The explicit use of theory in implementation research may support a better understanding of the generalisability and replicability of implementation interventions [17]. The MRC framework emphasises that the development, evaluation and implementation of health care interventions requires a strong theoretical foundation [15].

We have selected Normalisation Process Theory (NPT) [18,19] to provide a theoretical underpinning to this study. NPT can be used as a theoretical framework to identify factors that promote and inhibit the routine incorporation of complex interventions into everyday practice. It explains how these interventions work in practice (or fail to), looking not only at early implementation, but beyond this to the point where an intervention becomes so embedded into routine practice that it 'disappears’ from view (i.e., it is normalised) [20]. We will use NPT as an explanatory framework to explore the implementation process and to guide interviews with NHS staff. The use of NPT will provide a transferable understanding of the success or failure of the *HeLP-Diabetes* integration into routine NHS practice, providing learning opportunities for future studies.

## Methods/Design

### Design

This will be a longitudinal cohort study using qualitative and quantitative methods.

### Setting

The study will be conducted in GP surgeries, community diabetes clinics and hospital based diabetes clinics within two inner city London boroughs: There are approximately 80 GP surgeries, two services providing a range of community diabetes clinics and three hospitals running diabetes clinics within these two boroughs. Our aim is to make *HeLP-Diabetes* available to up to 30 GP surgeries and all diabetes clinics. Within these boroughs there are 16,223 people on GP registers with a recorded diagnosis of diabetes [21]. Since T2DM accounts for 90% of all diabetes cases, there are up to 14,601 potential users of *HeLP-Diabetes* within these two boroughs.

### Procedure

The use of *HeLP-Diabetes* will be offered to GP surgeries and diabetes clinics free of charge for the duration of the study (30 months). *HeLP-Diabetes* will be made available to batches of GP surgeries and clinics (approx. 2 or 3 at a time). This will allow learning and experience gained from the implementation at each batch of sites to be transferred to subsequent ones. Once organisations have agreed to use *HeLP-Diabetes* they will be asked to opt-in to the research evaluation study. Organisations who opt-out of the research evaluation will still be free to use *HeLP-Diabetes* without participation in the research.

Staff working in services that agree to use *HeLP-Diabetes* will be asked to recommend its use to all suitable patients with T2DM and refer them to a training appointment with a researcher or a member of the clinical team. Suitable times to recommend the use of *HeLP-Diabetes* may include at annual diabetes reviews and when a patient is newly diagnosed. Staff will also be encouraged to maintain engagement with *HeLP-Diabetes* by encouraging patients to use it at appropriate times during routine consultations.

During training appointments patients will be registered on the *HeLP-Diabetes* site and shown how to use it. Each appointment will be tailored to the specific needs of the patient and will focus on an aspect of diabetes self-management important to the patient. At the end of the training appointment patients will be invited to take part in the research study evaluating the implementation of *HeLP-Diabetes*. Patients who agree to take part in the evaluation study will be asked to sign a consent form after reading the participant information sheet and having the opportunity to ask any questions. Patients who opt-out of the research evaluation will still be free to use *HeLP-Diabetes* without participation in the research.

### Ethical consideration

Ethical approval for this study was gained from the NRES Committee East Midlands-Leicester ref: 13/EM/0033.

### Recruitment

Recruitment will happen at both organisational and patient level.

Both organisations and patients will be recruited firstly to use *HeLP-Diabetes* and secondly to take part in the research evaluation of the implementation.

#### Organisational

Strategies being explored to recruit organisations to use *HeLP-Diabetes* include engagement of the Clinical Commissioning Groups (CCG), direct contacts with hospital and community based clinics and the assistance of the North Central London Research Consortium (NoCLOR) to recruit GP surgeries.

Staff in participating services who opt in to the research evaluation will be invited to take part in research activities on an opportunistic basis throughout the study period.

#### Patient

Once a GP surgery or diabetes clinic has agreed to use *HeLP-Diabetes,* recruitment to use *HeLP-Diabetes* at the patient level will begin. Health professionals in participating GP surgeries and clinics will be asked to recommend the use of *HeLP-Diabetes* to all patients with T2DM who meet the inclusion criteria and to refer them to a training appointment. At the end of training appointments, eligible patients will be asked to participate in the research study evaluating the implementation of *HeLP-Diabetes*.

### Sample

#### Staff

A range of staff from GP surgeries and diabetes clinics including consultants, salaried doctors, junior hospital doctors, general practice partners, general practice managers, practice nurses, community nurses, hospital diabetes nurses and reception staff will be recruited to participate in the research evaluation study. The aim is to represent a range of perspectives of the staff who contribute to the care of patients with T2DM on the implementation of *HeLP-Diabetes*.

#### Patient

To use *HeLP-Diabetes* and participate in the evaluation study, patients must be 18 or above, registered at participating GP surgeries, community diabetes clinics or hospital based diabetes clinics and have a diagnosis of T2DM. Patients will be excluded from the research evaluation if they are unable to provide informed consent, e.g. due to psychosis, dementia or severe learning disabilities, are terminally ill (life expectancy less than 12 months), unable to use a computer due to physical or mental impairment or possess English language skills insufficient to use the intervention.

### Implementation plan

An implementation plan has been devised by the research team which will be offered to all services. This plan includes the following steps; an initial meeting at the GPsurgery or clinic to explain the new *HeLP-Diabetes* service, a demonstration of *HeLP-Diabetes*, training for staff who will be using *HeLP-Diabetes* top-up training and support as necessary, training patients to use *HeLP-Diabetes*, follow up contact with patients, continued monitoring and feedback to staff, and the provision of training materials to staff and patients. Each GP surgery and diabetes clinic will be invited to work with researchers to tailor the implementation plan to the individual setting.

The researcher will spend time with staff to implement *HeLP-Diabetes* and to provide support and facilitate patient training appointments. Formal feedback from interviews with staff and patients and informal feedback such as lessons learned from training staff and patients will be used by the researcher to develop and refine the implementation procedures. The iterative design will allow the implementation plan to be tested out on a small scale, the researcher to learn from the experience and modifications to be made to plan before testing it on a another small number of services. This method will allow for difficulties and challenges to be explored and addressed, which may otherwise be missed by widespread implementation. Through the process of modifying the implementation plan based on feedback from patients and staff and by allowing the implementation plan to be tailored to individual sites, we hope to increase buy-in and ownership of *HeLP-Diabetes* by staff and reduce barriers to successful implementation.

### Outcomes

The study objectives will be addressed in the following ways.

a. To describe the uptake and use of *HeLP-Diabetes* by NHS services and patients.

The number of GP surgeries and diabetes clinics *HeLP-Diabetes* is offered to, and the numbers who take it up will be recorded. To describe the types of services that take up *HeLP-Diabetes* we will collect data including: the number of patients registered at the surgery or who attend the clinic (overall numbers and those with T2DM), socio demographics of the patient population and the number of staff. For GP surgeries we will also collect: Quality and Outcomes Framework (QOF) achievement and whether it is a training or non-training practice.

The number of patients who register on *HeLP-Diabetes* will be recorded and patient registration data will be used to describe the characteristics of patients who sign up. This data will include; year of birth, gender, ethnicity, the surgery or clinics they attend, duration of diabetes, existence of complications, how diabetes is currently managed; what help the patient would like with self-management, smoking status, internet access (home/public) and use of computer (basic/intermediate/advanced).

Data on *HeLP-Diabetes* use will be recorded automatically by the website software and Google Analytics and will include number of logins, length of time patients use *HeLP-Diabetes* per visit, pages that are visited and those that are not, and the tools, assessments, educational modules that are used and how often.

b. To identify factors which inhibit or facilitate implementation.

Semi-structured interviews will be conducted with key informants, both patients and health professionals and will be focussed on exploring the barriers and facilitators to implementation. The researcher will also keep detailed notes on the implementation process and document any challenges and successes.

c. To identify the resources needed for effective implementation.

A proforma will be used to collect quantitative data on the resources spent on implementing *HeLP-Diabetes*, which are predicted to be largely made up of staff and researcher time. Semi-structured interviews will be conducted with key informants, both patients and health professionals, to investigate the resources needed for effective implementation.

d. To explore the effects of *HeLP-Diabetes* on self-reported patient outcomes.

Patient participants will be asked to complete two self-report measures. The first will be the Problem Areas in Diabetes (PAID) scale [22] which is a health related quality of life and emotional distress measure. It is comprised of 20 items focusing on areas that cause difficulty for people living with diabetes including; social situations, food, friends and family, diabetes treatment, emotions, relationships with healthcare professionals and social support. The second measure will be the Diabetes Management Self-Efficacy Scale (DMSES-UK) [23] which measures the individual’s efficacy expectations for engaging in 20 diabetes self-management activities, for example, taking daily exercise and keeping to a healthy eating plan when away from home. A sample size of 100 patients will detect a 3.5 point difference in the mean PAID score from baseline and a 9 point difference in the mean DMSES score from baseline. These calculations are based on 90% power (α = 0.05).

Additional self-report measures may be introduced to new cohorts of patients in order to test developing hypotheses generated by interviews with patients and staff. For example, if a theme emerges from the interviews suggesting that using *HeLP-Diabetes* may increase patients’ levels of physical activity, we may administer a physical activity self-report measure to a new cohort of patients at baseline, 3 months and 12 months to quantitatively explore this. Power and sample size will be calculated for each new measure prior to it being administered.

### Data collection

After consenting to take part in the evaluation research study, patient participants will be allocated a unique study ID number and complete baseline measures. Participants will be asked to provide their email address, postal address and contact number for data collection purposes. They will be sent a link in an email to a web-based case report form which will host the data collection measures. The data entered into this form will be anonymous and only linked to the patient by the unique ID number. Participants will be asked to complete socio-demographic information at baseline and self-report measures at baseline, 3 months and 12 months. Socio-demographic information will be collected to describe the sample, and to compare intervention use and self-report measure data.

Both patient and health professional participants will be asked to take part in semi-structured interviews. Each participant may be interviewed several times throughout the course of the study. We will sample until saturation is reached. In order to capture a breadth of experiences interviews will be conducted with patients from a range of ethnic backgrounds, ages, socio-demographic statuses, length of time with diabetes, treatment types, and associated complications, as well as a range of experience with computers and the internet. Similarly, to capture a range of staff experiences, we will seek to interview consultants, salaried doctors, junior hospital doctors, general practice partners, general practice managers, practice nurses, community nurses, hospital diabetes nurses and reception staff from GP surgeries and diabetes clinics. It is likely that there will be different levels of engagement with *HeLP-Diabetes* and with the evaluation research study between different members of staff and between different services and sites. Therefore, not all interviews will be face to face; initial interviews and follow-up encounters with some participants may be short, highly focused telephone interviews. These will minimise the demand on participants, and is likely to be much more cost effective. However, more in-depth, semi structured interviews will be conducted with participants wherever possible. Participants may be sampled purposefully at times to ensure that we have captured a range of experiences and opinions.

Data on intervention use will be recorded automatically by the intervention software and Google Analytics and will be downloaded into a spread sheet. Data on the type of GP surgeries and diabetes clinics that take up *HeLP-Diabetes* will be collected from Population Manager Data, Public Health Observatories [21] data and a data collection form to be completed by the Practice Manager or Clinic Lead at each site. The time spent by individuals involved in the implementation of *HeLP-Diabetes* will be recorded on a proforma.

### Anaylsis

The proportion of GP surgeries and clinics offered *HeLP-Diabetes* who adopt it, the number of patients who register on *HeLP-Diabetes*, the socio-demographic characteristics of the patients who take part in the evaluation study and the characteristics of the clinics and GP surgeries that adopt *HeLP-Diabetes* will be described using descriptive statistics. SPSS statistical software [24] will be used for coding and analysing these data. Descriptive statistics will also be used to explain how patients make use of *HeLP-Diabetes* including counts of pages accessed, number of logins, the times of use, the duration of use and the patterns of use by different users. This data will be recorded automatically by the website software. The time and other resources expended on implementing *HeLP-Diabetes* will be recorded on a proforma and these will be collated on a spread sheet and data described descriptively.

The cost of health professional input from the GP surgeries and clinics will be based on national average rates using Personal Social Services Research Unit estimates. Estimates of the additional time required from the general practice and clinic staff where *HeLP-Diabetes* is implemented will be calculated from responses in the qualitative interviews. Resources required in training individual patients to use *HeLP-Diabetes* will be estimated.

Participant scores on the PAID and DSMSE-UK scales will be compared statistically at the different time points using a repeated measures analysis of variance test using time as the independent variable. If a statistically significant effect is found post-hoc tests will be used to explore this further. Follow up PAID and DSMSE-UK scores will be adjusted for initial scores and other baseline covariates including age, gender, and duration of diabetes. Group effects including gender and age will be assessed using mixed-between within subjects analysis of variance.

With respondents’ consent, all semi-structured interviews will be audio-recorded, transcribed (and edited to ensure anonymity of respondent) and data will be subjected to formal analysis. Patient and staff interviews will be analysed inductively to identify themes and categories, a process of constant comparison will be used to establish analytical categories. Atlas. ti [25] data collation and management software will be used to aid data management and analysis.

## Discussion

This study will explore what happens when a new internet-based self-management intervention for patients with T2DM is introduced into existing NHS services. Quantitative data will describe the use of *HeLP-Diabetes* by NHS organisations and patients and allow us to determine the socio-demographic characteristics of users and the type of service that take up *HeLP-Diabetes.* We will determine the cost of implementation and the resources needed for successful implementation. Qualitative data will allow us to understand the implementation process and explore barriers and facilitators to implementing *HeLP-Diabetes* into existing services. We will explore the possible impact of *HeLP-Diabetes* use on patient-reported health-related quality of life, emotional wellbeing and self-efficacy to self-manage T2DM. This study will produce findings useful for those embarking on the implementation of an internet based resource within the NHS.

Translating research into routine clinical practice is often a difficult task. Implementation research is often focused on the early stages of the implementation process [26] however, there is also a need to examine later stages of implementation, including the integration of innovations into the norms and practices of organisations. Exploring the implementation of *HeLP-Diabetes* with a longitudinal study over 30 months will provide an opportunity to examine issues and challenges that emerge during the many stages of implementation including the less researched later stages.

There are several strengths of the design of this study. Data on the implementation of *HeLP-Diabetes* will be collected from staff across diabetes services and from a range of staff within these services. This will include both management and front-line staff as these groups may have very different viewpoints and perceptions of organisational change [27].

The iterative design of the study will allow for the implementation plan to change and develop in line with experiential learning and feedback from those involved in the implementation process. This is important in creating a final implementation procedure that is fit for purpose and effective. The mixed method design will aid the process of exploring the impact of *HeLP-Diabetes* on patient outcomes. By conducting interviews with patients throughout the study, emerging themes pertaining to the possible benefits of *HeLP-Diabetes* can be explored in greater detail with further interviews. We can also test emerging hypotheses about the mechanisms of action borne out of the interviews by administering further self-report measures to new cohorts of patients at baseline, 3 month and 12 month follow up.

The study faces some potential methodological and practical challenges. The study is limited to two inner London boroughs; the patient population and the services within which we are seeking to implement *HeLP-Diabetes* which may limit the generalizability of findings. In terms of studying the patient experience of *HeLP-Diabetes*, only the views of those patients who agree to take part in the research evaluation will be included. The views of those who don’t use *HeLP-Diabetes* and those using it but not taking part in the research study will not be represented.

The need for patients to attend a training appointment to use *HeLP-Diabetes* may be a potential bottle-neck to uptake. However, we believe that this tailored appointment will ensure that patients can use *HeLP-Diabetes* optimally. A research question to be addressed within this study is what is the most effective and viable way to ensure *HeLP-Diabetes* is a useful, effective and sustainable resource for patients and the evaluation of this training appointment will be central to that.

It is likely that internet-based health interventions will become increasingly widespread, not just for diabetes but for other long term physical conditions and mental health problems. There is a need to understand the implementation process of these interventions including the uptake by health organisations and patients, the facilitators and barriers to use and the resources needed for successful implementation and normalization. This study will offer a contribution to this.

## Abbreviations

CCG: Clinical Commissioning Group; DMSES-UK: Diabetes management self efficacy scale; GP: General practice; NHS: National Health Service; NICE: National Institute for Health and Clinical Excellence; NPT: Normalisation process theory; PAID: Problem Areas in Diabetes; T2DM: Type 2 diabetes mellitus

## Competing interests

The authors declare that they have no competing interests.

## Authors’ contributions

All authors have made substantial contributions to conception and design of this study. JR developed the manuscript draft. All authors have read and given final approval of the version to be published.

## Pre-publication history

The pre-publication history for this paper can be accessed here:

http://www.biomedcentral.com/1472-6963/14/51/prepub

## References

[B1] Diabetes UK Diabetes in the UK 2010: key statistics on diabetes 2010 [Accessed on Dec 16, 2012]. Available from: http://www.diabetes.org.uk/Documents/Reports/Diabetes_in_the_UK_2010.pdf

[B2] Kanavos P van den Aardweg S Schurer W Diabetes expenditure burden of disease and management in 5 EU countries 2012 London: LSE Health [Accessed on Dec 16, 2012] Available from: http://www.lse.ac.uk/LSEHealthAndSocialCare/research/LSEHealth/MTRG/LSEDiabetesReport26Jan2012.pdf

[B3] Ellis SE Speroff T Dittus RS Brown A Pichert JW Elasy TA Diabetes patient education: a meta-analysis and meta-regression Patient Educ Couns 2004 14 97 105 10.1016/S0738-3991(03)00016-8 14729296

[B4] Deakin T McShane CE Cade JE Williams RDR Group based training for self-management strategies in people with type 2 diabetes mellitus Cochrane Database Syst Rev 2005 14 CD003417 15846663 10.1002/14651858.CD003417.pub2

[B5] Davies MJ Heller S Skinner T Campbell M Carey M Cradock S Effectiveness of the diabetes education and self management for ongoing and newly diagnosed (DESMOND) programme for people with newly diagnosed type 2 diabetes: cluster randomised controlled trial BMJ 2008 14 491 10.1136/bmj.39474.922025.BE 18276664 PMC2258400

[B6] Nicolucci A Cavaliere D Scorpiglione N Carinci F Capani F Tognoni G A comprehensive assessment of the avoidability of long-term complications of diabetes. A case-control study. SID-AMD Italian Study Group for the Implementation of the St. Vincent Declaration Diabetes Care 1996 14 927 933 10.2337/diacare.19.9.927 8875084

[B7] National Diabetes Audit: National Diabetes Audit 2010-2011 Report into the Data Quality of Diabetes Structured Education 2012 Accessed on [Dec 16, 2012] Available from: http://www.hqip.org.uk/assets/NCAPOP-Library/NCAPOP-2012-13/Diabetes-Audit-Report-10-11-StructuredEducation-pub-2012.pdf

[B8] Bennett G Glasgow R The delivery of public health interventions via the internet: actualizing their potential Annu Rev Public Health 2009 14 273 292 10.1146/annurev.publhealth.031308.100235 19296777

[B9] Murray E Burns J See TS Lai R Nazareth I Interactive health communication applications for people with chronic disease Cochrane Database Syst Rev 2005 14 CD004274 16235356 10.1002/14651858.CD004274.pub4 PMC13184810

[B10] Jackson CL Bolen S Brancati FL Batts‒Turner ML Gary TL A systematic review of interactive computer‒assisted technology in diabetes care J Gen Intern Med 2006 14 2 105 110 16390512 10.1111/j.1525-1497.2005.00310.x PMC1484664

[B11] Pal K Eastwood SV Michie S Farmer AJ Barnard ML Peacock R Wood B Inniss JD Murray E Computer‒based diabetes self‒management interventions for adults with type 2 diabetes mellitus Cochrane Database Syst Rev 2013 14 CD008776 23543567 10.1002/14651858.CD008776.pub2 PMC6486319

[B12] Minet L Moller S Vach WR Wagner L Henriksen JE Mediating the effect of self-care management intervention in type 2 diabetes: a meta-analysis of 47 randomised controlled trials Patient Educ Couns 2010 14 1 29 41 10.1016/j.pec.2009.09.033 19906503

[B13] Norris SL Lau J Smith SJ Schmid CH Engelgau MM Self-management education for adults with type 2 diabetes: a meta-analysis of the effect on glycemic control Diabetes Care 2002 14 7 1159 1171 10.2337/diacare.25.7.1159 12087014

[B14] Haas L Maryniuk M Beck J Cox CE Duker P Edwards L Fisher EB Hanson L Kent D Kolb L National standards for diabetes self-management education and support Diabetes Care 2013 14 Supplement 1 S100 S108 23264420 10.2337/dc13-S100 PMC3537270

[B15] Craig P Dieppe P Macintyre S Michie S Nazareth I Petticrew M Developing and evaluating complex interventions: the new Medical Research Council guidance BMJ 2008 14 a1655 10.1136/bmj.a1655 18824488 PMC2769032

[B16] Eccles MP Armstrong D Baker R Cleary K Davies H Davies S An implementation research agenda Implement Sci 2009 14 18 10.1186/1748-5908-4-18 19351400 PMC2671479

[B17] Public Health Observatories Health Profile 2012- Islington & Health profile 2012- Camden 2012 [Accessed on Dec 16, 2012] Available from: http://www.apho.org.uk/resource/item.aspx?RID=117231

[B18] May CR Mair F Finch T MacFarlane A Dowrick C Treweek S Development of a theory of implementation and integration: Normalization Process Theory Implement Sci 2009 14 29 10.1186/1748-5908-4-29 19460163 PMC2693517

[B19] May C Finch T Implementing, embedding, and integrating practices: an outline of Normalization Process Theory Sociology 2009 14 535 554

[B20] Murray E Treweek S Pope C MacFarlane A Ballini L Dowrick C Normalisation process theory: a framework for developing, evaluating and implementing complex interventions BMC Med 2010 14 63 10.1186/1741-7015-8-63 20961442 PMC2978112

[B21] Health Profile 2012- Islington & Health profile 2012- Camden: Public Health Observatories [Accessed on Dec 16, 2012] Available from: http://www.apho.org.uk/resource/item.aspx?RID=117231

[B22] Polonsky WH Anderson BJ Lohrer PA Welch G Jacobson AM Aponte JE Assessment of diabetes-related distress Diabetes Care 1995 14 754 760 10.2337/diacare.18.6.754 7555499

[B23] Bijl J Poelgeest-Eeltink A Shortridge-Baggett L The psychometric properties of the diabetes management self-efficacy scale for patients with type 2 diabetes mellitus J Adv Nurs 1999 14 352 359 10.1046/j.1365-2648.1999.01077.x 10457237

[B24] IBM Corp IBM SPSS Statistics for Windows, Version 20.0 2011 Armonk, NY: IBM Corp

[B25] Muhr T “Atlas ti (Version 6.2. 16)[computer software] 2011 Berlin: Atlas ti GmbH

[B26] Fixsen DL Naoom SF Blase KA Friedman RM Implementation Research: a synthesis of the literature 2005 Tampa, FL: University of South Florida, Louis de la Parte Florida Mental Health Institute, The National Implementation Research Network (FMHI Publication #231) 119

[B27] Hasson H Gilbert-Ouimet M Baril-Gingras G Brisson C Vézina M Bourbonnais R Montreuil S Implementation of an organizational-level intervention on the psychosocial environment of work J Occup Environ Med 2012 14 85 91 10.1097/JOM.0b013e31823ccb2f 22193115

